# Prevalence and severity of preoperative anemia, and associated factors among orthopedic patients at public comprehensive referral hospitals, Northwest Ethiopia 2024: multi- center cross-sectional study

**DOI:** 10.1186/s13741-025-00578-w

**Published:** 2025-11-13

**Authors:** Atalay Eshetie Demile, Habtu Tsehayu Bayu, Endale Gebreegziabher Gebremedhn

**Affiliations:** 1 Department of Anaesthesia, School of Medicine, College of Medicine and Health Sciences, Bahir Dar University, Bahir Dar, Ethiopia; 2 Department of Anaesthesia, School of Medicine, Gondar College of Medicine and Health Sciences, University of Gondar, Gondar, Ethiopia

**Keywords:** Preoperative anemia, Prevalence of preoperative anemia, Orthopedic surgery

## Abstract

**Background:**

Preoperative anemia is a major clinical problem that increases perioperative patient morbidity and mortality. Additionally, preoperative anemia causes hemodynamic instability, delayed recovery after anaesthesia and surgery, prolong length of hospital stay and increases risk of postoperative infection. However, the prevalence of preoperative anemia remains unexplored in conflict affected areas.

**Objective:**

To assess prevalence and severity of preoperative anemia and associated factors among orthopedic patients in Bahir Dar city Comprehensive Referral Hospitals, Northwest Ethiopia, 2024.

**Methods:**

Multi-center cross-sectional study was conducted. Data was collected using questionnaire and checklist. All consecutive scheduled emergency and elective patients were included in the study. Data was transformed from Epi data to SPSS and logistic regression analysis was done. Both crude and adjusted odds ratio were used to assess the strength of association. Hosmer –Lemeshow test and multi collinearity were checked. Variables with a p-value of less than 0.05 were considered as statistically significant.

**Results:**

A total of 820 patients were included in this study with a response rate of 99.9%. Prevalence of preoperative anemia was 46.7% (n = 383/820) with (95%, CI = 0.33353- 0.5987) in this study. Emergency orthopedic cases (AOR = 3.014, CI = 2.480–5.717), traumatic related orthopedic cases (AOR = 2.01, CI = 1.480- 3.21), repeated history of anesthesia and orthopedic surgery (AOR = 3.11, CI = 1.480- 3.54), presence of coexisting diseases (AOR = 1.501, CI = 1.002- 3.74) and preoperative blood loss greater than 500 ml (AOR = 3.001, CI = 2.012- 5.104) were associated with preoperative anemia.

**Conclusion and recommendation:**

The prevalence of preoperative anemia among orthopedic patients was high in the study area. Orthopedic patients should be screened for preoperative anemia routinely. Additionally, nutritional and iron therapy should be given for mild to moderate anemia. Moreover, blood transfusion should be considered for patients with severe anemia.

##  Background

World Health Organization defines anemia as a decrease in hemoglobin (Hb) levels to less than 12 g/dL for women who are not pregnant and 13.0 g/dL for males (Abdullah, et al., 2017). Nearly one-third of people worldwide suffer with preoperative anemia, which is the most often occurring kind of anemia (Almac, et al., 2014). Patients undergoing major orthopedic surgery frequently experience preoperative anemia, which affects up to 20% of cases (Beyable, et al., 2022).

Anemia is linked to higher rates of morbidity and mortality in women, stunted growth in children, increased the risk infections, and a decrease in productivity due to reduced work capacity (Bisbe, et al., 2008, Burns, et al., 2019). Preoperative anemia is prevalent in individuals in orthopedic surgery and a separate risk factor for perioperative morbidity and mortality (Bisbe, et al., 2008).

Pre-operative anemia is independently linked to a higher risk of unfavorable outcomes such as longer hospital admissions and critical care stays, perioperative problems, and higher patient mortality and morbidity (Burton, et al., 2018, Diress, and Ayele, 2023). Additionally, preoperative anemia raises the risk of oxygen depletion, increases morbidity and mortality in orthopedic surgery, and is associated with poor postoperative outcomes (Diress, and Ayele, 2023).

Preoperative anemia is a potent predictor of the need for blood transfusions, but transfusions are linked to longer hospital stays, more complicated surgeries, and higher rates of morbidity and death (Ferraris, et al., 2012). Moreover, preoperative anemia is quite common although there is considerable improvement in the techniques to lessen reliance on allogeneic blood transfusion as a treatment for surgical anemia, raising questions about the necessity of liberal or preventive allogeneic blood transfusion (Gangat, and Wolanskyj, 2013).

Age, gender, comorbidities, surgical indication, lifestyle and socioeconomic status affect the prevalence of pre-operative anemia (Goodnough, et al., 2011, Hong, et al., 2017). A person’s age, gender, residential elevation above sea level (altitude), smoking habit, and stage of pregnancy all affect their specific physiological needs. The most common cause of anemia worldwide is thought to be iron deficiency, but other nutritional deficiencies like vitamin deficiency, acute and chronic inflammation, parasitic infections, and inherited or acquired disorders that affect the synthesis of haemoglobin (Jans, et al., 2014).

Generally, preoperative anemia in orthopedic patients remains a major clinical problem as it imposes several adverse effects such as increase perioperative patient morbidity and mortality, intraoperative haemodynamic instability, delayed recovery from anaesthesia, increase the risk of postoperative infection and death (Beyable, et al., 2022, Burton, et al., 2018, Diress, and Ayele, 2023). However, the prevalence of preoperative anemia in orthopedic patients remains unexplored in conflict affected areas. Our aim is to study the prevalence of preoperative anemia and associated factors among orthopedic patients in Bahir Dar City public comprehensive referral hospitals. We hypothesized that the prevalence of preoperative anemia among orthopedic patients will be high in conflict affected areas.

## Objectives

### General objective

To assess prevalence and severity of preoperative anemia, and associated factors among orthopedic patients in Bahir Dar City Comprehensive Hospitals, Norwest Ethiopia, 2024.

### Specific objectives

To determine the prevalence preoperative anemia among orthopedic patients.

To determine severity of preoperative anemia among orthopedic patients.

To identify potential risk factors for preoperative anemia among orthopedic patients.

## Methods

### Study design, setting and period

A multi-center cross-sectional study was conducted from May 1/05/2024 to July 30/07/2024 in selected two comprehensive and specialized referral hospitals in Amhara National Regional State, Northwest Ethiopia.

**Tibebe Ghion comprehensive specialized hospital:** Found at Bahir Dar city which has seven general operation rooms, and two orthopedic operation rooms. On average around 250 orthopedic patients are being operated per month according to hospital reports.

**Felege hiwot referral comprehensive hospital:** Found at Bahir Dar city which has seven general operation rooms, and two orthopedic operation rooms. On average around 220 orthopedic patients are being operated per month according to hospital reports.

### Source population

All surgical patients admitted emergency and elective orthopedic wards of study area.

### Study population

All surgical patients how underwent surgery at orthopedic operation room at study area.

### Eligibility criteria

#### Inclusion criteria

Both elective and emergency orthopedic patients who scheduled for surgical services.

#### Exclusion criteria

Orthopedic surgical patients who have not preoperative hemoglobin investigation and patients who have history of chronic anemia.

### Sample size and sampling technique

The sample size was calculated using single proportion formula by taking a proportion of 50% and assuming 95 confidence interval and 5% margin of error.

**Fig. 1:**
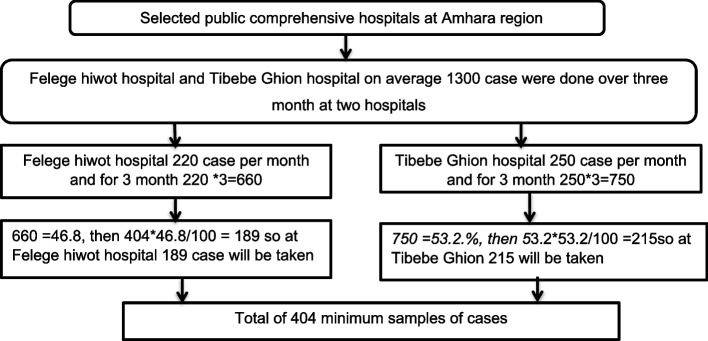
Sample size allocations for each hospital

$n=\frac{{\left(Z{}^{\alpha}\!\left/\!{}_{2}\right.\right)}^{2}p\left(1-p\right)}{{d}^{2}}$, where n = is the desired sample size; z = z –score value of 95% confidence interval is 1.96; *p* = population proportion of 50% (0.5) and *d* = degree of accuracy desired or marginal error is 5% (0.05));

Then n = 1.962 * 0.5*0.5/(0.05)^2^, n = 384. In addition, with a 5% non-response rate, the final sample size is 404 patients from two hospitals (Fig. 1).

Sampling technique**:** All consecutive orthopedic patients who scheduled for surgery during the study period were included.

### Operational definition

**Blood loss estimation:** preoperative blood loss was estimated by reviewing the records from the patients’ charts.

**Anemia:** According to WHO criteria, anemia was defined by an Hb concentration < 12 g/dL for women or < 13 g/dL for men. Severe anemia was defined by an Hb concentration < 10 g/dL (Killip, et al., 2007).

**Iron deficiency anemia**: defined as a ferritin concentration < 30 ng/mL, as this cut-off point seems to be more appropriate for the diagnosis of iron deficiency in the elderly (Kotze, et al., 2012).

**Vitamin deficiency anemia**; Serum levels of vitamin B_12_ and folic acid were measured by radio immunoanalysis. According to laboratory reference values, vitamin B_12_ deficiency was defined as a serum concentration < 270 pg/mL (200 pmol/L) and folic acid deficiency by a serum concentration < 3 ng/mL (Lane, and Rojas-Fernandez, 2002).

**Anemia of chronic disease:** Patients with anemia and creatinine > 17 mg/dL and/or LESR > 30 mm/hour were considered to have anemia of chronic disease (ACD). Serum creatinine levels were measured by an Olympus 5200 multi-analyzer (Lasocki, et al., 2015).

### Variables of the study

#### Dependent variable

Anemia (yes/no).

#### Independent variables

Age, sex, nutritional status (normal, undernutrition and overweight or obesity), Body mass index/or BMI (normal, underweight, overweight, obese, morbid obese), American Society of Anesthesiologists (ASA) status, type of surgery (upper vs lower extremity surgery), comorbidity (yes, no), Duration of illness, urgency of procedures (emergency vs elective) reason of operation (trauma vs non-trauma), amount of preoperative blood loss, history of repeated anesthesia and surgical exposure,

### Data collection methods and quality control

Data were collected using interviewer administered questionnaire and chart review. Data were collected during preoperative assessment at preoperative anesthesia waiting room. Questionnaires was prepared in English format and translated back to Amharic version. Chart review to see the hemoglobin level on patient chart and diagnosis anemia according to WHO with dichotomous outcome (ye/no). After written informed consent was obtained, the trained data collector collected the data and the investigators assess the overall completeness of the data. To check the quality of questionnaires pretest was done on 10% of the total sample.

### Statistical analysis

After we collected data, the questionnaire paper was checked manually for completeness and then it was coded at Epi data version 4.6 and was transformed from Epi data to SPSS and analyzed using SPSS Version 26. Descriptive statistics were carried out and the results were presented using text, tables, and graphs. Assumption of binary logistic regression was checked including chi-square test and continuous variables were normally distributed presented using mean and standard division and non-normally distributed data presented using median and interquartile range. The goodness of fit of model was checked using (Hosmer and Lemeshow (p-value = 0.79), omnibus test of model coefficient (p-value = 0.001)). Variance inflation factor and tolerance was used to test multicollinearity (p-value = 0.98, 1.026). Logistic regression analysis was performed to determine the independent variables which have association with the dependent variable. Both crude and adjusted odds ratio were used to assess the strength of association between dependent and independent variables. Those variables with p-value less than 0.2 from the bivariable regression analysis were fitted for the multivariable logistic regression analysis. At 95% confidence interval a p-value of less than 0.05 was considered as statistically significant.

#### Ethical consideration

Ethical clearance was obtained from the institutional review board of the college of medicine and health science, School of Medicine, Bahir Dar University ethical review committee with ethical registration number of (protocol number 906/2024). Permission letter was also obtained from the two hospitals.Written informed consent was obtained from each study subject after a clear explanation during the preoperative period about the objective, purpose and as they have the right to refuse to participate in the study. Confidentiality was ensured by avoiding personal identifiers and locking the questionnaire and checklist. We informed the responsible surgeons and anaesthetists about those patients with moderate and severe anemia to treat anemia ahead of operation.

## Results

### Socio-demographic characteristics of the study participants

A total of 821 patients who underwent orthopedic surgery were included in the study. However, 1 patient was excluded due to incomplete data. Therefore, the final sample size for this study was 820 patients with a response rate of 99.9%. Of 820 patients, 450 were operated at Tibebe Ghion specialized hospital (54.9%), and 370 at Felege hiwot specialized hospital (45.1%) (Table 1). Among the participants, 308 (37.6%) were females and 512 (62.4%) were males. The average age of the study participants was 32.05 ± 12.7, with the majority (60%) were under 30 years old (Table 1).

**Table 1:** Socio demographic characteristics of the study participants (N = 820)

Variables	Category			Frequency(n)	Percentage (%)
sex	Female	Female		308	37.6
	Male	Male		512	62.4
Age	Minimum	Maximum	Range	Mean	St. deviation
	2	87	85	32.05	12.7
BMI	< 18.5			20	2.4
	18.5–24.9			510	62.2
	25–29.9			219	26.7
	> 30			71	8.7
Hospitals where operation done	TGCSH			450	54.9
	FHCSH			370	45.1

Key: BMI = Body Mass Index

FHCSH = Felege Hiwot Comprehensive Specialized Hospital and TGCSH = Tibebe Ghion Comprehensive Specialized Hospital

### Patient characteristics

Of 820 study participants, majority 555 (67.7%) were ASA II, 233 (28.4%) ASA I and 32 (3.9%) ASA III respectively (Table 2). Most patients (n = 411, 50.1%) had not history of previous anesthesia and surgical exposure, whereas 409 (49.9%) had history of previous anesthesia and surgical exposure (Table 2).

**Table 2:** Factors related with patients (N = 820)

Variables	Category	Frequency (n)	Percentage (%)
ASA Classification	ASA I	233	28.4
	ASA II	555	67.7
	ASA III	32	3.9
History of repeated anesthesia and orthopedic surgical exposure	Yes	409	49.9
	No	411	50.1

Key: ASA = American Society of Anaesthesiology

### Factors related with the orthopedic surgery

Trauma was the most common cause of orthopedic operation (n = 706, 86.1%) followed by osteomyelitis (n = 63, 7.7%) and malignancy (n = 51, 6.2%) respectively. The majority of the orthopedic procedures were emergency surgery (n = 472, 57.6%), whereas 348 (42.4%) operations were elective orthopedic surgery. Debridement and irrigation was the most common orthopedic procedure (n = 290, 35.4%) followed by sign nailing (n = 270, 32.9%), open reduction and internal fixation (n = 100, 12.2%), plating (n = 96, 11.7%) and external fixation (n = 64, 7.8%). Additionally, of 820 study participants, most of them have no coexisting diseases. Only 121 (14.8%) had coexisting disease. Three hundred and ninety patients (47.6%) were had less than 500 ml preoperative blood loss, whereas four hundred and thirty (52.4%) had preoperative estimated blood loss of greater than 500 ml (Table 3).

**Table 3:** Factors related with surgery (N = 820)

Variables	Category	Frequency (n)	Percentage (%)
Diagnosis	Trauma	706	86.1
	Malignancy	51	6.2
	osteomyelitis	63	7.7
Urgency of operation	Elective	348	42.4
	Emergency	472	57.6
Orthopedic surgical procedure	Debridement and irrigation	290	35.4
	sign nailing	270	32.9
	open reduction and internal fixation	100	12.2
	plating	96	11.7
	external fixation	64	7.8
coexisting diseases	yes	121	14.8%
	no	695	84.8%
preoperative blood loss	< 500	390	47.6%
	> 500	430	52.4%

### Prevalence and severity of preoperative anemia

The overall prevalence of preoperative anemia was 46.7% (n = 383/820) with (95%, CI = = 0.3353- 0.5987) in this study. Among the overall prevalence of anemia moderate anemia was most common (n = 186, 48.6%) followed by mild anemia (n = 162, 42.3%) and the prevalence of severe anemia was low (n = 35, 9.1%). Additionally, among anemic patients (n = 169, 44.1%) of them were given blood transfusion. Of those, 98% were severely anemia, but the rest of them were managed by fluids. With regarding the prevalence of preoperative anemia among individual hospitals; at Tibebe Ghion comprehensive specialized hospital 47.6% (n = 214/450) (CI = 43.1**–**51.9) and at Felege Hiwot comprehensive specialized hospital 48.1% (n = 178/370) (CI = 45.8**–**59.1) respectively (Table 4). With regard to the prevalence of preoperative anemia there was no significance difference between the two hospitals (Table 4).

**Table 4:** Prevalence and severity of preoperative anemia (N = 820)

Variables		Category		Frequency(n)	Percentage (%)	
Is there anemia according to WHO		No		437	53.3	
		Yes	Mild	162	42.3	46.7
			Moderate	186	48.6	
			Sever	35	9.1	
		Is there was any transfusion	Yes	169	44.1	
			no	214	55.9	
Name of hospitals		Anemic	Non-anemic			
1	TGCSH	214(47.6%)	236(52.4%)	450(54.9%)		
2	FHCSH	178(48.1%)	192(51.9%)	370(45.1%)		

Key; FHCSH = Felege Hiwot Comprehensive Specialized Hospital

TGCSH = Tibebe Ghion Comprehensive Specialized Hospital

WHO = World Health Organization

### Factors associated preoperative anemia

The variables that had a p value of less than 0.2 from bivariable logistic regression include the sex of the patients,age of the patients,traumatic related orthopedic producers, reputed exposure of anesthesia and surgery, presence of coexisting diseases, urgency of the procedure,ASA status of the patients, and preoperative blood lose greater than 500 ml. Among those variables, traumatic related orthopedic procedures, repeated exposure of anesthesia and surgery, presence of co-existing diseases, urgency of the procedure and preoperative blood loss greater than 500 ml had significant association with prevalence of preoperative anemia from the multi variable logistic regression with p value < 0.05 (Table 5).

**Table 5:** .Factors associated with prevalence of preoperative anemia (N = 820)

Variables		Preoperative anemia according to WHO		COR(95% CI)	AOR (95% C.I)	*P* value
		No(n)	Yes (n)			
Urgency of operation	Emergency	273(57.8%)	199(42.2%)	3.93(2.94–6.75)	3.014(2.480–5.717)	.0001
	Elective	90(25.9%)	258(74.1%)	(1)		
Cause of orthopedic procedures	Traumatic	490(69.4%)	216(30.6%)	4.2(2.31–3.92)	2.01(1.480–3.21)	.0017
	Non-traumatic	40(35.1%)	74(64.9%)	(1)		
Repeated exposure of anesthesia and orthopedic surgery	Yes	281(68.7%)	128(31.3%)	3.82(2.11–14.8)	3.110(1.48–3.54)	.000258
	No	150(36.5%)	261(63.5%)	(1)		
Presence of coexisting diseases	Yes	72(59.5%)	49(40.5%)	2.74(1.8–18.23)	1.501(0.872–2.62)	.00014
	No	244(34.9%)	455(65.1%	(1)		
Preoperative blood loss	> 500 ml	283(72.6%)	107(27.4%)	4.94(3.08–25.29)	3.001(2.012–5.104)	.00001
	< 500 ml	150(34.9%)	280(65.1%)	(1)		

Key; COR = Crude Odds Ratio, AOR = Adjusted Odds Ratio, C.I = Confidence Interval

Interval

Multivariable logistic regression analysis showed that emergency orthopedic surgical patients are 3 times more likely to be anemic as compared to elective surgery (AOR = 3.014,CI = 2.480–5.717). Traumatic related orthopedic procedures could increase of being anemic by two times as compared with non- traumatic related orthopedic procedures (AOR = 2.01,CI = 1.480- 3.21). Additionally, repeated exposure of anesthesia and orthopedic surgery increased the risk to be anemic three times as compared to single orthopedic procedures (AOR = 3.11,CI = 1.480- 3.54). Moreover, the presence of co-existing diseases increased the chance of being anemic for orthopedic patients by one and half times (AOR = 1.501, CI = 1.002- 3.74).and preoperative blood loss greater than 500 ml increased the occurrence of preoperative anemia among orthopedic patients by three times (AOR = 3.001, CI = 2.012- 5.104) (Table 5).

## Discussion

This study determined the prevalence of preoperative anemia and contributing factors for anemia in orthopedic patients. The findings of this study will help to the develop strategies that will enable to prevent preoperative anemia such as developing preoperative anemia screening programme and implementation of preoperative optimization methods such as nutritional, vitamin B12 and iron supplementation and blood transfusion as appropriate. These strategies will reduce perioperative patient morbidity, mortality, length of hospital stay and postoperative infections.

This study showed that the prevalence of preoperative anemia among orthopedic patients in Amhara National Regional Sate Bahir Dar City public comprehensive specialized hospitals was 46.7%. Out of this, 186 (48.6%) patients developed moderate anemia, 162 (42.3%) patients developed mild anemia and 35 (9.1%) patients developed severe anemia. The risk factors analyzed by multi-variable logistic regression are emergency orthopedic cases, trauma orthopedic cases, repeated history of anesthesia and orthopedic surgery, presence of co-existing diseases and preoperative blood loss greater than 500 ml.

In this study, the prevalence of preoperative anemia was 46.7%. This finding was high as compared with the study done in Debre Tabor university among elective orthopedic procedures with the prevalence of preoperative anemia of 24.1% (Marsicano, et al., 2018) and a study conducted at the University of Gondar specialized hospital among adult major elective surgery with the prevalence of preoperative anemia of 36.8% (Matsuda, et al., 2007). Additionally, our finding was high as compared with other previous studies. Most studies found that the prevalence of preoperative anemia among orthopedic patients with a ranged of 7% to 36.8% (Matsuda, et al., 2007, Muñoz, et al., 2015, Muñoz, et al., 2015b). The high prevalence of preoperative anemia in our study could be due to large number of emergency trauma cases were included in the study.

In this study, moderate anemia was most common (48.6%) followed by mild anemia (42.3%) and the prevalence of severe anemia was low (9.1%). This study revealed that the prevalence of preoperative anemia among each comprehensive hospitals was (46.7%) at Tibebe Ghion specialized comprehensive hospital and 48.1% at Felege Hiwot specialized comprehensive hospital. There was no significant difference between hospitals. The possible reason could be the two hospitals are fund in the same town and have comparable level of cases flow and similar level of care for orthopedic patients.

This study found that being emergency surgery increased the likelihoods of preoperative anemia among orthopedic patients rate by three times compared to elective procedures. This finding is high as compared with a previous study conducted for elective surgery at University hospital in Ethiopia (Matsuda, et al., 2007). Additionally, our finding was similar with another study conducted in Ethiopia among elective orthopedic procedures (Marsicano, et al., 2018). This could be due to the fact that we included emergency trauma patients and trauma is the most common cause of bleeding which in turn results in mild to severe anemia. Additionally, there could be lack of adequate resuscitation efforts in case of massive trauma causality and the presence of inadequate blood and blood products. Moreover, in emergency situations, most patients arrive to the hospitals late and the quality of preoperative care might be substandard as the emergency patients are mainly managed by practioners like residents and students which might contributes for the increments of preoperative anemia.

In this study, the presence of co-existing disease increased the chance of occurrence of preoperative anemia among orthopedic patients. The presence of chronic disease like;cancer, chronic kidney disease, heart failure, autoimmune diseases (such as rheumatoid arthritis, lupus, and inflammatory bowel disease) and diabetes could cause anemia and if co-existing disease superimposed with trauma, it may increase the occurrence of anemia (Marsicano, et al., 2018). Our study revealed that the presence of coexisting disease increased the chance of occurrence of preoperative anemia by 1.5 times. A previous study suggested that the presences’ of coexisting disease like cancer, kidney disease and patients with retroviral infections with anti-retroviral therapy increased the chance of developing anemia (Marsicano, et al., 2018). The presence of coexisting disease increased the occurrence of preoperative anemia due to the fact that, coexisting diseases can suppress the bone marrow function and decrease activations of erythropoietin steam cells and malnutrition which leads to deficiency of iron and some important vitamins.

Additionally, this study found that repeated exposure of anesthesia and orthopedic surgery is a risk factor for the occurrence of preoperative anemia. History of repeated exposure of anesthesia and orthopedic surgery increased the likelihood of developing anemia by three times as compared with those who has single anesthesia and surgery exposure. This finding is consistent with a previous study conducted in Debre Tabor teaching hospital showed that the occurrence of anemia among elective orthopedic patients was highly associated with previous history of surgery and anesthesia (Matsuda, et al., 2007). Our finding is also in line with a study conducted in Singapore (Musallam, et al., 2011). The possible reason could be that repeated surgery and anesthesia on postoperative period reduces the production of erythropoiesis which is important for the production of red blood cells. Additionally, surgery-associated inflammation causes anemia. Moreover, liberal fluid administration may cause dilutional anemia in the postoperative period.

Moreover, this study showed that trauma related orthopedic procedure increased the probability of occurrence of preoperative anemia among orthopedic patients by two times as compared with non-trauma orthopedic procedures. Our finding was in line with a previous study done in Spain where they fund that perioperative blood loss associated with preoperative anemia in patients with substantial long bone fracture and pelvic fracture (in hip or knee replacement surgery) (Naghi Abedini, et al., 2019). This is also supported by another study done in Melbourne, Australia on prevalence and causes of preoperative anemia (Bisbe, et al., 2008). This may due to the reason that trauma causes tissue damage and penetrating trauma occurs when an object penetrates the patient’s body, resulting in tearing or cutting the blood vessels and blunt trauma. This kind of trauma happens when a body part collides with something else, usually at high speed. Blood vessels inside the body are torn or crushed either by shear forces or a blunt object. Altogether, causes bleeding which results in anemia.

Furthermore, this study found that preoperative blood loss greater than 500 ml increased the likelihoods of having anemia by three times as compared with blood loss less than 500 ml. Our finding supported by a study done in California on the effect of estimated blood loss on preoperative anemia (Sim, et al., 2017). This clinical problem could be a major concern in resource limited sittings where there will be low level of preoperative anemia screening and optimization. Additionally, the availability of limited blood supplies and blood products enforced healthcare providers to rely mainly on fluid resuscitation which could result in dilutional anemia.

Older patients, sex and patients with higher ASA class are at high risk of preoperative anemia (World Health Organization 2014). But these clinical factors had not association with preoperative anemia in the current study. This could be due to the fact that the majority of patients were young age group (60%) and ASA 1–2 (96.1%). The aforementioned points indicate that preoperative anemia in orthopedic patients should be prevented by establishing routine preoperative screening programme. Additionally, those patients with anemia should be optimized by providing nutritional, vitamin B12 nd iron supplementation and blood transfusion as appropriate.

## Strength and limitation of the study

The strength is that the study this is a multicenter and with large sample size of the study. The limitations of this study include lack of data on some variables such as hemoglobin measurement timing relative to surgery and ferritin and vitamin B12. Additionally, this is a cross-sectional study and potential selection and blood loss estimation biases and patients with chronic anemia were excluded.

## Conclusions and recommendations

The prevalence of preoperative anemia in orthopedic surgical patients was high in Bahir Dar City comprehensive specialized hospitals. Emergency orthopedic surgery, traumatic related orthopedic procedure, repeated exposure of anesthesia and orthopedic surgery, presence of coexisting diseases and preoperative blood loss greater than 500 had strong association with preoperative anemia in orthopedic patients. We suggest that orthopedic patients should be screened routinely for anemia during the preoperative period. Additionally, nutritional, vitamin B12 and folate and iron supplementation and blood transfusion should be considered as appropriate for anemic patients. Moreover, we suggest further research on the effect of preoperative anemia on the perioperative outcome of orthopedic surgical patients.

## Data Availability

All the raw data for this paper are available upon reasonable request to the corresponding author.

## Abbreviations

ASA: American Society of Anaesthesiology
BMI: Body Mass Index
FHCSH: Felege Hiwot Comprehensive Specialized Hospital
TGCSH: Tibebe Ghion Comprehensive Specialized Hospital
WHO: World Health Organizations
